# Optimization of the color masking and coating unit operations for microencapsulating ferrous fumarate for double fortification of salt

**DOI:** 10.1007/s13197-022-05426-z

**Published:** 2022-04-19

**Authors:** Oluwasegun Modupe, Yao Olive Li, Levente L. Diosady

**Affiliations:** 1 Department of Chemical Engineering and Applied Chemistry, University of Toronto, 200, College Street, Toronto, ON M5S 3E5 Canada; 2 Department of Nutrition & Food Science, California State Polytechnic University, Pomona, USA; 3 School of Chemistry and Materials Science, RIT, One Lomb Memorial Drive, Rochester, NY 14623 USA

**Keywords:** Double fortified salt, Ferrous fumarate, Iodine, Microencapsulation, Pan coating, Soy stearin, Titanium dioxide

## Abstract

A new coating formulation was developed to eliminate the factor that caused black spots on the iron premix surface, used for making Double Fortified Salt. The formulation is a suspension of titanium dioxide in soy stearin, prepared with ethanol and dichloromethane and applied with a glass sprayer and pan coater. 0–20% ^w^/_w_ titanium dioxide was suspended in 10% ^w^/_w_ soy stearin/hydroxypropyl methylcellulose. Coating with a suspension of 15% ^w^/_w_ TiO_2_ in 10% ^w^/_w_ soy stearin ensured that all the TiO_2_ adheres to the premix surface, giving no chance for the recycling of iron contaminated TiO_2_, which caused the black spot. The new coating formulation ensured that over 90% iodine in Double Fortified Salt was retained after 6 months at 45 °C, 60–70% RH. The whiteness of the premix (L* = 86.4) matched the Double Fortified Salt whiteness (L* = 86.8). Thus, making the new coating method as effective as the previous in desirable characteristics. More so, the new coating method simplifies the existing method by merging the previous color masking, and double coating steps into one step.

## Introduction

Salt became a choice vehicle for delivering micronutrients to a large population, given the global use and impact of iodized salt in reducing the global prevalence of iodine deficiency (Andersson et al. 2012). Hence, many studies have been conducted to add other micronutrients to salt (Ranganathan 1992; Zimmermann et al. 2002; Zimmermann et al. 2004; Vinodkumar and Rajagopalan 2009; Modupe and Diosady 2021). Iron is predominant among the other micronutrients added to salt because of the very high global prevalence of iron deficiency (Stoltzfus 2003). However, the direct addition of iron to iodized salt accelerated iodine loss in the salt. Hence, several technologies were developed to prevent iodine loss in salt due to the addition of iron. These technologies included the use of stabilizers and absorption enhancers and iron microencapsulation. The technologies minimized the adverse interaction between iron and iodine in the salt, aided the bioavailability of iron, and minimized iron's adverse impact on the fortified salt's organoleptic properties (Wegmüller et al. 2003; Rao 1994; Li et al. 2010). A comparative study carried out on these technologies showed that iron (iron premix) made by extrusion-based microencapsulation is better than the rest in terms of sensory properties, iron bioavailability, and iodine stability (Andersson et al. 2008). The encapsulant forms a physical barrier between iron and iodine and masks the ferrous fumarate (iron) undesired color (brown).

Several attempts were made to formulating iron premix for the double fortification of salt with iron and iodine. The formulation involves iron particle agglomeration, color masking, and surface over-coating. A one-step approach to all these processes, using a pan coater, fluidized bed, or spray dryer, was attempted as the first-generation technology (Oshinowo et al. 2012; Yusufali 2001; Romita et al. 2011). The iron premix samples obtained from these processes were nonuniform in shape and size. These reasons made the Research Group at the University of Toronto to ultimately settle for second-generation technology, which is a cold-forming extrusion-based agglomeration of ferrous fumarate, followed by separate unit operations for cutting and size matching, color masking, and the agglomerates' surface-coating. The four main steps involved in making the iron agglomerate, iron premix, can be optimized.

Li et al. (2011) and Yadava et al. (2012) optimized this technology at the laboratory-scale. Both studies proposed the use of durum semolina as a binder and vegetable shortening as a lubricant for extrusion, titanium dioxide as a color masking agent, and hydroxypropyl methylcellulose (HPMC) as the surface over-coating material to partially or fully replace the hydrophobic coating (soy stearin) in the original formulation (in the first-generation technology). A dough made from ferrous fumarate, durum semolina, vegetable shortening, and water is extruded through an angel hair die. In the laboratory, the extrudate was cut with a coffee grinder; the color masking was achieved by dusting titanium dioxide on the surface of the premix in a beaker with a plastic spatula; the color-masked particle was coated with HPMC using a fluidized bed spray coater or coated with soy stearin using a pan coater.

The Li et al. (2011) and Yadava et al. (2012) studies led to an established lab-scale innovation for making a stable and bioavailable iron premix for the double fortification of salt with iron and iodine. The technology is easily adaptable to the traditional process of salt iodization. The process of making iron premix is an additional operation to the conventional process of making iodized salt. The technology was scaled up by several pilot testing trials, which eventually led to India's full commercialization (Jadhav et al. 2019).

The technology was slightly modified during the technology transfer based on the design and configurations available at JVS Food PVT Ltd, the pilot plant. At the plant, a spheronizer cut and polished the extrudate from cylindrical extrudate into sphere balls (300–700 µm). The round iron particles were then dried in a tray dryer to keep the moisture content at 5–10% ^w^/_w_ before the next step of sieving to collect uniform-sized iron balls. A drum coater, which is less sophisticated and lower-cost equipment than a fluidized bed spray coater, was used for color masking and coating. Unlike at the laboratory scale, the color masking and coating steps were intermittent because the drum coater was used for both processes at the pilot plant. TiO_2_ was dusted on the dried iron balls during the drum coater's tumbling operation as the desired amount of HPMC solution in a 1:1 ethanol and water solvent system (5% ^w^/_v_ for desirable viscosity) was sprayed on the color-masked iron balls. The intermittent application allowed the glue effect of HMPC solution to stick TiO_2_ powder on the surface of the iron balls in the drum. After 5% ^w^/_w_ HPMC has been applied, 5–10% ^w^/_w_ molten soy stearin was applied to finalize the coatings.

Due to the compressed air required for applying HPMC, some TiO_2_ is usually lost (being blown away). Therefore, about 25–35% ^w^/_w_ TiO_2_ is commonly used for color masking, but 15–20% ^w^/_w_ should sufficiently mask the extrudate's color. Before the final coat is applied, the excess TiO_2_ is removed by sieving. In contrast, the fluidized bed used at the laboratory scale has a suction facility that removes the excess TiO_2_. The excess amount of titanium dioxide used for color masking at the pilot plant triggered the reuse of TiO_2_ between batches to save operational costs.

During the initial trials for the technology scale, black spots were observed on the iron premix's surface. The excessive use of TiO_2_ led to its recycling between batches; the recycled TiO_2_ contaminated with iron particles from the iron extrudate caused black spots on the iron premix surface. The observed dark spots on the premix affected the acceptance and the stability of iodine in the salt. This study describes a novel coating approach that eliminated the factor, recycling TiO_2_ between batches, which led to the observed dark spots on the premix's surface. The new coating approach's impact on iron bioaccessibility and prevention of the adverse interaction between iron and iodine in Double Fortified Salt was also investigated.

## Materials and methods

### Materials

The ferrous fumarate (iron) extrudate used was obtained from *JVS Foods PVT. Ltd. (Jaipur, Rajasthan, India).* Soy stearin (SS), hydroxypropyl methylcellulose (HPMC), and titanium (IV) oxide, used for coating and color masking iron extrudate at the Food Engineering Laboratory at U of T, were obtained from *JVS Foods PVT. Ltd. (Jaipur, Rajasthan, India); Dow Chemical Company (Midland, Michigan USA);* and *ACROS Organics (Fair Lawn, New Jersey, USA),* respectively. Absolute ethanol and dichloromethane used for dissolving coating material were obtained from *Thermo Fischer Scientific (Mississauga, Ontario, Canada).* Nitric acid and hydrochloric acid, disodium EDTA, and iron standard reagent used in sample preparation and iron quantification were obtained from *Caledon Laboratory Ltd (Georgetown, Ontario, Canada), BioShop Canada Inc. (Burlington, Ontario, Canada), and Sigma–Aldrich Chem (Oakville, Ontario, Canada).* All chemicals used for the fortification of salt were food-grade, while those used for analysis were ACS grade.

### Methods

#### Effect of the amount of TiO_2_ and the method of applying TiO_2_ and coating material on the color of the premix

The optimum amount of TiO_2_ required for effective color masking was determined in the laboratory to prevent the use of an excessive amount of TiO_2_ (which resulted in its reuse). Pan coater, a 2-D version of drum coater used at the pilot plant, was used to apply the coating material on the iron extrudate. Pan coater was used because the fluidized bed initially used at the laboratory scale has a suction compartment that removes the blown away TiO_2_. Hence, the fluidized bed cannot quantify the actual amount of TiO_2_ required. Four different amounts of TiO_2_ were used (5, 10, 15, and 20% ^w^/_w_) for dusting iron extrudate, after which 5% ^w^/_w_ HPMC and 5% ^w^/_w_ soy stearin were applied by pan coating (double coating). Also, a fluidized bed was used to coat two control samples with 5% ^w^/_w_ HPMC. The samples were initially color masked with 12.5 or 25% ^w^/_w_ TiO_2_. After they were coated with HPMC, they were further coated with 5% ^w^/_w_ soy stearin by pan coating. A third control sample was not color masked with titanium dioxide but was coated with HPMC or soy stearin by pan coating. The premix's whiteness was an indicator for the use of an optimum amount of TiO_2_.

Titanium dioxide was dusted on the extrudate's surface in a beaker using a plastic spatula; coating materials (HMPC and soy stearin) were applied using a pan coater and a glass sprayer. HPMC (2% ^w^/_v_) dissolved in a 1:1 ethanol-dichloromethane solvent system was sprayed on the color-masked extrudate using a glass sprayer (atomizer) at about 3 ml/min. A hairdryer attached to the pan coater base aids the solvents' quick evaporation. The second two control samples were color masked extrudates and coated using a fluidized bed, as described by Li et al. (Li et al. 2011). The molten soy stearin (5% ^w^/_v_) dissolved in dichloromethane was also sprayed on the coated extrudate, such that the color masked extrudate was double coated with 5% ^w^/_w_ HPMC and 5% ^w^/_w_ soy stearin. The coated premix was spread on an aluminum foil sheet and air-dried for 2–3 days for complete solvent evaporation.

#### Effect of coating with a suspension of TiO2 in coating material solution

Even when pan coater and drum coater were used, a small amount of TiO_2_ was still blown away from the extrudate's surface due to compressed air used to apply the coating material's solution. The feasibility of coating with a suspension of TiO_2_ in the solution of coating material was evaluated to prevent the blowing away of loosely attached TiO_2_. Instead of having separate color masking and double coating unit operations, these operation units were merged in the new process. The brown iron extrudate was coated with a suspension of TiO_2_ in HPMC or soy stearin to ultimately coat the extrudate with 5–20% ^w^/_w_ TiO_2_ and 10% ^w^/_w_ HPMC or soy stearin. The TiO_2_ was thoroughly mixed with the solution of soy stearin or HPMC. Total solids (TiO_2_ + coating material), 3, 4, and 5% ^w^/_v_ and 2.5, 5, and 8% ^w^/_v_ were evaluated for having an effective suspension of TiO_2_ in HMPC and soy stearin, respectively. The extrudate was also coated with 10% ^w^/_w_ HPMC or soy stearin without TiO_2_ to determine the coating materials' contribution to the premix's whiteness. These premix samples' color was compared to some control samples made by having separate color masking and coating units. The same amounts of color masking and coating material were used in the control samples.

#### Evaluation of the effect of the loading capacity of pan coater on effective coating

Before coating with a suspension of TiO_2_ in coating material, the optimal ratio of the amount of premix to pan coater size was determined. Four different amounts (5, 10, 15, and 20 g) of premix were coated in a pan coater with a fixed size (Diameter = 16 cm). A suspension of 15% ^w^/_w_ TiO_2_ in 10% ^w^/_w_ soy stearin solution was used for coating the premix. The color and uniform distribution of the suspension were examined. An iPhone 8 Camera was used to capture the pictures of the premix samples in a photo box. The pictures were used to evaluate the distribution of the coating materials on the extrudate.

#### Determination of the total iron content in premix samples

The method described by Moldoveanu and Papangelakis (Moldoveanu and Papangelakis 2013) was adapted for quantifying total iron content in the premix. The premix samples (100 mg) were added into digestion vials. Aqua regia (3:1 molar ratio HCl: HNO_3_, 15 ml) was then added to the vial, and its cap tightened. The samples were digested with a microwave digester (ETHOS EZ, Milestone Inc.) for 2 h. The resulting solutions were poured into 50 ml volumetric flasks and made to the mark with 5% ^v^/_v_ HNO_3_. The solution was diluted at a ratio of 1:9 with 5% ^v^/_v_ HNO_3_. The sample's iron content was analyzed using an inductively coupled plasma optical emission spectrometer (Agilent Dual View 720).

#### Determination of the amount of iron on the surface of the premix

This method relied on the use of a solution of Na_2_EDTA, a good iron chelator. Na_2_EDTA (20 ml of 5% ^w^/_v_ adjusted to pH 7 with sodium hydroxide) was added to 100 mg iron premix samples into a 40 ml beaker. The mixture was stirred on a magnetic stirrer for 5 min and filtered with a syringe filter (0.45 µm). The filtrate was diluted at a ratio of 1:9 with 5% ^v^/_v_ HNO_3_. The resulting solution's iron content was then analyzed with an inductively coupled plasma optical emission spectrometer (Agilent Dual View 720). The result was expressed as a percentage of the total iron in the premix.

#### Evaluation of the surface morphology of the premix

The scanning electron microscope (SU-3500 VP SEM, Hitachi High-Technologies) was used to determine the premix's surface morphology. The premix was attached to an SEM specimen stub with a carbon conductive double-coated adhesive tape. Air was blown over the attached premix to remove any loose premix. Samples were examined, and micrographs were recorded at an acceleration voltage of 1.5 kV, with a working distance of 51 mm, under a high vacuum, as described by Singh et al. (2018). However, the sample was not gold-coated, as described by Singh et al. (2018), to make the surface defects more visible.

#### Determination of the densities of premix

An empty 25 ml scintillation vial was weighed (W_1_), then filled with the premix samples, and tapped until no apparent volume change was observed. The weight of the sample-filled vial was then recorded (W_2_). The vial was emptied and filled with water, and weighed (W_3_). The bulk density of the sample was then calculated according to Eq. 1:

1 $$\rho_{B} = \frac{{W_{2} - W_{1} }}{{W_{3} - W_{1} }} \times \rho_{W}$$

where ρ_B_ is the bulk density in g cm^−3^;

ρ_w_ is the water density = 1 g cm^−3^.

After the bulk density was determined as described, the void volume in the sample-filled flask was determined by a hexane dropwise addition. The weight of the flask was measured (W_4_), and Eq. 2 was used to calculate the particle density

2 $$\rho_{P} = \frac{{\left( {W_{2} - W_{1} } \right)}}{{\left( {\frac{{W_{3} - W_{1} }}{{\rho_{W} }}} \right) - \left( {\frac{{W_{4} - W_{2} }}{{\rho_{H} }}} \right)}}$$

where ρ_P_ is the particle density in g cm^−3^;

ρ_w_ is the water density = 1 g cm^−3^;

ρ_H_ is the hexane density = 0.66 g cm^−3^.

#### Evaluation of the sinking properties of the premix

Water (about 700 ml) in a 1000 ml beaker was stirred with a magnetic bar stirrer. A known number of the premix particles (50) was counted and gently added to the beaker's stirrer water. The number of premix particles still floating after 2 min was counted. To accurately count by visual inspection, the beaker was placed on a dark platform so that white floating premix can be easily seen and counted. The floating of more than two premix particles was judged as significant because that would keep more than 95% of the premix from being lost through floating and subsequently being washed away during cooking.

#### Estimation of the thickness of layers of materials in the premix

The impact of the amount of TiO_2_ used for color masking on the thickness of each layer of coating materials and TiO_2_ that make up the premix samples was evaluated. Three assumptions were made for estimating the thickness of the layers– that the particles were spherical, uniform distribution of the material in each layer, and no loss of material during premix production.

The estimation considered the proportion of each of the materials that made up the premix, their densities, and the particle's average weight. The total volume of core materials (ferrous fumarate, semolina, and fat) was calculated from each material's density and average mass of material used per premix. The total volume was obtained from the addition of the volumes of the core materials. The radius (r) was estimated from the total volume estimates. For calculating the thickness of additional layers (TiO_2_, HPMC, and soy stearin), each material's volume, as they were added to the core material, was added to the core's total volume. The radius of the new total volume was estimated as earlier described. The thickness of the material (T) was estimated based on the differential radii (Eq. 3). For instance, for the TiO_2_ layer, the volume of TiO_2_ used to mask the particle was added to the core materials' volume. The radius (r_1_, calculated from the core material volume) was subtracted from the radius (r_2_) of the added volumes to give the estimated thickness of the TiO_2_ layer in the premix. The same procedure was repeated for HPMC and soy stearin layers.

3 $$T = r_{2} - r_{1 }$$

#### Evaluating the mechanical strength of the premix coating

The premix and salt were added to a ribbon blender. They were mixed in the ribbon blender for 10 min. The premix was physically examined for falling apart of their coat, which was observed as dark spots on the premix.

#### Evaluation of the color of the premix

The L*a*b* color properties of premix samples were determined using a colorimeter (Chroma Meter CR-400/40, Konica Minolta Photo Imaging USA, Inc., Mahwah, NJ) as described by Modupe (Modupe 2020). The L*a*b color analysis was used to assess the whiteness of the premix as a yardstick for the impact of the amount of TiO_2_ and the coating method used on the effectiveness of color masking. The whiteness index (WI) and the color differences (∆E) of the premix were calculated as described by Khazaei et al. (2014).

#### In vitro iron bioaccessibility approximation

The iron premix disintegrates when DFS is used for cooking. However, the iron release profile from the premix coated with a suspension of TiO_2_ in soy stearin was compared with the iron premix color masked and coated with the previous method. As described by Modupe and Diosady (Modupe and Diosady 2021), the premix samples (100 mg) and then 250 ml 0.1 M HCl were added into 500 ml Erlenmeyer flasks. The flasks were placed inside a Cole-Parmer StableTemp Water bath (EW-14575–12) coupled with Cole-Parmer Polyscience Dual Action Shaker, set at 37 °C, 160 rpm for 2 h. For all premix samples, 1 ml of the solution was taken from the digestion tube at 30-min intervals. The withdrawn solution (1 ml) was mixed with 9 ml 5% ^v^/_v_ nitric acid. The solutions were filtered with a 45 µm syringe filter. The concentration of the iron in the solutions was measured using ICP-OES. The amount was presented as a percentage of the total amount of iron in the premix.

#### Formulation of fortified salt

A potassium iodate solution (5 ml, 3.37% ^w^/_v_) was sprayed on and mixed with a refined salt (2 kg) in a ribbon blender so that the concentration of iodine in the salt was 50 ppm (Modupe et al. 2021a, b). The salt was air-dried overnight and mixed with 10 g of iron premix so that the concentration of iron in the salt was 1000 ppm. Two iron premix samples were used- one was color masked with 15% ^w^/_w_ TiO_2_ and then coated with 10% ^w^/_w_ soy stearin, while the other was coated with a suspension of TiO_2_ (15% ^w^/_w_) in soy stearin (10% ^w^/_w_). Formulated iodized salt was used as the control sample. Iodine was analyzed in the salt sample, as described by Modupe et al. (2019), immediately the salt was formulated and after 6 months of storage at 25, 35, 45 °C, 60–70% RH.

### Statistical analysis

All experiments were conducted with at least triplicates. The results were calculated and expressed as a means ± standard deviation for each of the measurements. The data were subjected to one-way ANOVA using SPSS software, and the differences between means were considered significant at *P* < 0.05.

## Results and discussion

During commercial-scale production of iron premix, black spots were observed on the surface of some premix particles. The dark spots on the white iron premix were a severe problem as color is one factor that drives consumer acceptance of food products (Dias et al. 2012; Clydesdale 1993). The use of recycled TiO_2_ contaminated with iron was responsible for the black spot observed. The problem can be solved by using the right amount of titanium dioxide for color masking. However, the compressed air used to apply the coating material would force the pilot plant operators to use an excessive amount of TiO_2_. Hence, in the new approach, TiO_2_ was suspended in a coating material solution such that both the TiO_2_ and the coating material were applied together like paint on the surface of the premix.

### Optimizing the amount of titanium dioxide required for color masking

The first step in designing the newly formulated lab coating formulations was to determine the optimal amount of TiO_2_ required for color masking. The premix samples were double coated with 5% ^w^/_w_ HMPC and 5% ^w^/_w_ soy stearin after being color masked. The whiteness of the premix presented as L* values increased with the increase in TiO_2_ used for color masking (from 5 to 20% ^w^/_w_). However, the magnitude of increase dropped when the amount of TiO_2_ used was increased from 15 to 20% ^w^/_w_. Also, the lightness of the premix that was color masked with 15% ^w^/_w_ TiO_2_ (L* = 88.8 ± 2.4) was not significantly different from the color of Double Fortified Salt reported by Zimmermann et al. (2003) (L* = 86.8 ± 2.6). Hence, 15% ^w^/_w_ was chosen as the optimal TiO_2_ required for color masking.

After they were double-coated with HPMC and soy stearin, the whiteness of the premix samples showed that a significant amount of TiO_2_ was blown away during fluidized bed (as judged from the whiteness of the premix in Table 1 and given that TiO_2_ was used as the whitening agent). The amount of TiO_2_ lost was higher with the use of a fluidized bed than a pan coater, justifying the preferred use of a pan coater over a fluidized bed in this study.

**Table 1:** Color characteristics of iron premix

TiO_2_ (% ^w^/_w_)	Method	L*	WI	∆E
12.5	Fluidized Bed	75.6 ± 0	75.5 ± 0.1	N/A
25	Fluidized Bed	76.2 ± 0.7	76.1 ± 0.7	0.7 ± 0.6
0	Pan Coater	51.8 ± 4.9	50.5 ± 5.1	N/A
5	Pan Coater	75.1 ± 1.8	75.0 ± 1.8	25.7 ± 2.9
10	Pan Coater	84.5 ± 3.6	84.5 ± 3.6	34.7 ± 1.1
**15**	**Pan Coater**	**88.8 ± 2.4**	**88.7 ± 2.4**	**38.7 ± 2.2**
20	Pan Coater	90.9 ± 2.0	90.9 ± 2.0	40.8 ± 2.3

The premix samples were color masked with TiO_2_ and double-coated with HMPC and soy stearin. The premix samples varied in the amount of TiO_2_ used for color masking the premix and the method used to coat the premix. As the amount of TiO2 used increased for pan coating, the color difference (∆E) significantly increased until 15 to 20% ^w^/_w_ TiO where the increased ∆E was not significant. L^*^ (+ = lighter; −  = blacker); a^*^ (+ = red; −  = green); b^*^ (+ = yellow; − = blue); ∆E: difference in color between and premix standards (color masked with 12.5% ^w^/_w_ TiO2 and coated with fluidized bed or not color masked); WI: whiteness index; the values were averages of 3 replicates ± standard deviation

### Impact of the amount of titanium dioxide used for color masking on the size and effective coating of iron premix

Since the amount of coating material used in the premix was kept at 10% ^w^/_w_ (5% HPMC and 5% soy stearin) as established by Yadava et al. (2012), the amount of TiO_2_ used for color masking would impact the thickness of the coating material layer around the premix. The mass and densities of the materials used to encapsulate the iron core of the premix were critical in estimating the volume and the thickness of the encapsulants' layers.

**Fig. 1:**
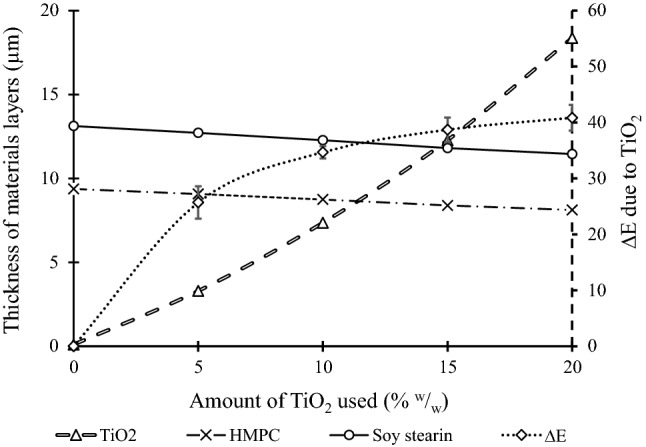
The effect of the amount of TiO_2_ used for color masking on the whiteness of premix and thickness of double HPMC and soy stearin coat layers of the premix. This figure showed the impact of the amount of TiO2 used for colr masking on the thickness of the double coating layers of the premix and the premix whiteness. The ∆E is against the right Y-scale, while the others are against the left Y-Scale. As the TiO_2_ used was increased, the thickness of the TiO_2_ layer increased while the thickness of HPMC and soy stearin layers in the premix decreased. Hence, the thickness of the TiO_2_ layer has a negative correlation with the thickness of HPMC and soy stearin layers. However, ∆E has a positive correlation with the amount of TiO_2_ used for color masking. For ∆E, the values were averages of 5 replicates ± standard deviation; the differences between means were considered significant at P < 0.05

Titanium dioxide, being denser than the other materials used in formulating iron premix, significantly impacted the premix's mass. Increasing the amount of TiO_2_ used for color masking increased its layer's thickness in the premix; specifically, a more significant difference between r_2_ and r_1_ when the iron core's size was the same. This increase impacted the size of the premix particle. The average particle sizes were 721.8, 722.8, 726.2, 732.0 and 749.6 µm for particles color masked with 0, 5,10, 15, 20% ^w^/_w_ titanium dioxide, respectively. Since the amount of HMPC and soy stearin used was kept constant (5% ^w^/_w_ each), their layers' thickness was reduced as the amount of TiO_2_ increased. So, the amount of TiO_2_ negatively correlates with the HPMC or soy stearin layer's thickness (Fig. 1). Increasing the amount of TiO_2_ from 15 to 20% ^w^/_w_ did not significantly improve the premix's whiteness. More so, this increase reduced the thickness of the HPMC and soy stearin layer, which may result in a compromised coating. The proposal of 15% ^w^/_w_ TiO_2_ as being the optimal amount of TiO_2_ required for coating was further justified with this result.

The amount of titanium dioxide used significantly but not appreciably impacted the particle and bulk densities of the premix. The premix has particle densities 1.00 ± 0.00, 1.06 ± 0.01, 1.19 ± 0.02 and 1.24 ± 0.01 and bulk densities 2.02 ± 0.01, 2.08 ± 0.01, 2.10 ± 0.01 and 2.18 ± 0.07 for premix color masked with 5, 10, 15, 20% ^w^/_w_ titanium dioxide, respectively.

### Determination of the optimal ratio of the amount of premix to pan coater size

The use of 15% ^w^/_w_ TiO_2_ cannot prevent TiO_2_ from being blown from the extrudate's surface by the compressed air required for drum coating operation. If this loss of TiO_2_ is not prevented, the operators will still use excess TiO_2_. The feasibility of coating premix with a suspension of TiO_2_ in HPMC or soy stearin was evaluated as a potential path to prevent the excessive use or the reuse of TiO_2_. If successful, the initially separated color masking and coating unit operations of the process would be merged; so that both the whitening and coating agents are applied once, like white paint on the surface of the extrudate.

**Fig. 2:**
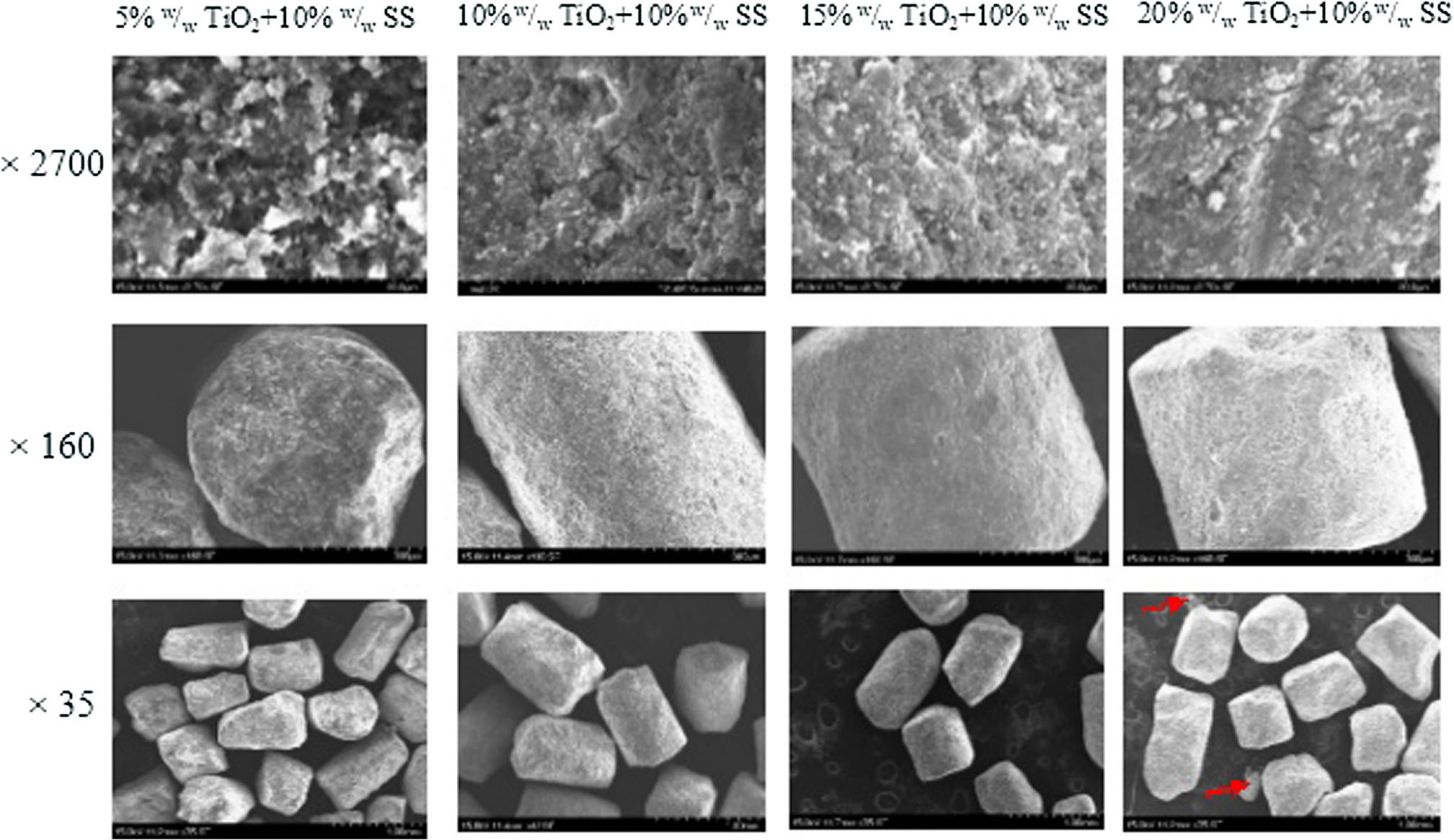
The effect of the amount of extrudate in a pan coater (diameter = 16 cm) on a uniform and effective coating of the iron extrudate. The L* values for the premix samples are 66.46, 79.48, 86.37, and 90.83, respectively. When 5 g of premix was coated in a pan coater (with 16 cm diameter), most of the droplets of TiO_2_ suspended in soy stearin solution fell on the exposed pan coater base instead of the extrudate. As the amount of extrudate coated was increased, more proportion of the pan coater base was covered with the extrudates until 20 g extrudate was used. With 20 g, layers of extrudates built up in the pan coater; some extrudate surfaces did not receive enough suspension droplets

Before coating with a suspension of TiO_2_ in coating material, an optimized loading capacity of the drum/pan coater (the amount of extrudate per batch of coating operation given the coater's fixed size), which is essential to achieving an effective coating, was determined. The optimal loading capacity was 15 g extrudate in a pan coater with a 16 cm diameter and 5 cm height. A lesser loading capacity resulted in most of the coating blend falling on the pan coater base surface rather than the extrudate surface, while higher loading capacity resulted in fewer extrudates not effectively coated. Hence, a lower loading capacity resulted in a uniform coating but with a lesser degree of whiteness, while a higher loading capacity resulted in a nonuniform coating (Fig. 2). The optimal loading capacity of the drum coater used at the pilot plant has been established.

### Feasibility of coating with a suspension of titanium dioxide in coating material solution

Soy stearin contributes more to the premix's whiteness than HPMC (Table 2). Not surprisingly, this is owing to the opacity of solidified fat (soy stearin) compared to the transparent film formed by HPMC. Also, coating with a suspension of TiO_2_ in soy stearin solution (concentration of total solids in the suspension = 5% ^w^/_v_:) was better than coating with a suspension of TiO_2_ in HPMC solution (concentration of total solids in the suspension = 4% ^w^/_v_) in terms of ease of flow material through the nozzle of the glass sprayer. Hence, a suspension of TiO_2_ in soy stearin solution was used in subsequent sets of the coating. In terms of the whiteness of the premix, there was no significant difference between coating with a blend coating system (a suspension of TiO_2_ in soy stearin solution) and a separate coating system of color masking first (with 15% ^w^/_w_ TiO_2_) followed by coating with 10% ^w^/_w_ soy stearin. For premix coated with a suspension of TiO_2_ in soy stearin solution, the amount of TiO_2_ used corresponded to the degree of whiteness of the premix. However, the impact (∆WI*) decreases as the amount of TiO_2_ increases such that increasing the concentration of TiO_2_ from 15 to 20% did not essentially improve the whiteness of the premix when the premix samples were visually inspected and compared with the color of salt. Hence, a suspension of 15% ^w^/_w_ TiO_2_ in the 10% ^w^/_w_ soy stearin solution is the optimal coat. The lightness of the premix (L* = 86.4) was very close to the lightness of the Double Fortified Salt reported by Zimmermann et al. (2003) (L* = 86.8).

**Table 2:** Feasibility of coating with a suspension of TiO_2_ in soy stearin solution

Coating Material	L*	a*	b*	WI
10% ^w^/_w_ HPMC (without TiO_2_)	37.4 ± 0.9^a^	11.5 ± 0.1^f^	16.0 ± 0.4^f^	35.6 ± 0.7^a^
10% ^w^/_w_ Soy stearin (without TiO_2_)	56.7 ± 4.8^b^	5.0 ± 1.4^e^	7.9 ± 1.0^e^	50.5 ± 5.1^b^
5% ^w^/_w_ TiO_2_ + 10% ^w^/_w_ SS Blend	64.4 ± 0.3^c^	0.3 ± 0.2^d^	− 4.2 ± 0.2^a^	64.2 ± 0.2^c^
10% ^w^/_w_ TiO_2_ + 10% ^w^/_w_ SS Blend	76.8 ± 0.2^e^	− 1.2 ± 0.2^b^	− 3.7 ± 0.5^a^	76.4 ± 0.1^e^
15% ^w^/_w_ TiO_2_ + 10% ^w^/_w_ SS Blend	86.4 ± 0.3^f^	− 1.5 ± 0.1^a,b^	-0.7 ± 1.0^c^	87.0 ± 0.2^f^
20% ^w^/_w_ TiO_2_ + 10% ^w^/_w_ SS Blend	91.9 ± 1.4^ g^	− 1.7 ± 0.4^a^	− 1.8 ± 1.1^a,b^	92.1 ± 0.9^ g^
10% ^w^/_w_ TiO_2_ + 10% ^w^/_w_ HPMC Blend	60.3 ± 1.0^b^	1.7 ± 0.2^e^	0.8 ± 0.2^d^	60.2 ± 0.9^b^
15% ^w^/_w_ TiO_2_ + 10% ^w^/_w_ HPMC Blend	74.0 ± 1.3^d^	− 0.5 ± 0.3^c^	− 2.5 ± 0.1^b^	73.4 ± 0.7^d^
Color masked(15%^w^/_w_ TiO_2_); Coated(10%^w^/_w_ SS)	88.8 ± 3.5^f,g^	− 0.6 ± 0.3^c^	0.2 ± 1.3^c,d^	88.7 ± 2.4^f^

Premix samples were coated with varying proportion of TiO_2_ and soy stearin in suspension. The whiteness index of the premix increased as the amount of TiO_2_ in the suspension increased. L^*^ (+ = lighter; −  = blacker); a^*^ (+ = red; − = green); b^*^ (+ = yellow;  −   = blue); HPMC: hydroxypropyl methylcellulose; SS: soy stearin; the values were averages of 5 replicates ± standard deviation; the differences between means were considered significant at *P* < 0.05

The coats (5–15% ^w^/_w_ TiO_2_ and 10% ^w^/_w_ soy stearin) on the premix can withstand the mechanical friction of mixing with salt in a ribbon blend, unlike the 20% TiO_2_ and 10% soy stearin coat. There was no noticeable dark spot on the four premix samples' surface, except in premix coated with a suspension of 20% ^w^/_w_ TiO_2_ in soy stearin solution after the premix samples were mixed with salt. This result shows that there is an allowable limit to the ratio of TiO_2_ to soy stearin. If this ratio is exceeded, there may be a need to add a layer of soy stearin.

There is a tendency for soy stearin to make the premix float; hence, the premix density was investigated. The soy stearin to TiO_2_ ratio in the suspension tends to impact the premix's density (Table 3). There was a significant increase in the particle and bulk density as the proportion of TiO_2_ increases. This observation is not surprising as TiO_2_ had the highest particle density of all materials used in formulating the premix. However, all four premixes' bulk density was greater than 1 g.cm^−3^, the water density; hence, all the premix sank when added to water. The observed densities of the premix samples were higher than those reported by Yadava et al. (2012) except for the density of the premix coated with a suspension of 5% ^w^/_w_ TiO_2_ in soy stearin. This is consistent with the densities of the premix samples reported by Modupe et al. (2021a, b). The loss of TiO_2_ from the extrudate when Yadava et al. (2012) used a fluidized bed spray coater may be responsible for this.

**Table 3:** Properties of premix coated with a suspension of TiO_2_ in soy stearin solution

Coating Material	Bulk density (g/cm^3^)	Particle density (g/cm^3^)	Shedding off (TiO_2_)	Amount of Iron Exposed (%^w^/_w_)
5% ^w^/_w_ TiO_2_ + 10% ^w^/_w_ SS Blend	1.03 ± 0.01_a_	1.88 ± 0.03^a^	Not observed	0
10% ^w^/_w_ TiO_2_ + 10% ^w^/_w_ SS Blend	1.08 ± 0.00^a^	1.96 ± 0.03^a^	Not observed	0
15% ^w^/_w_ TiO_2_ + 10% ^w^/_w_ SS Blend	1.16 ± 0.00^b^	2.15 ± 0.02^b^	Not observed	0
20% ^w^/_w_ TiO_2_ + 10% ^w^/_w_ SS Blend	1.20 ± 0.00^b^	2.18 ± 0.00^b^	Observed	7.54 ± 0.08

The amount of TiO_2_ in the suspension impacted the densities of the premix. When 20% TiO_2_ was used, the capacity of soy stearin to hold TiO_2_ in position on the premix's surface was compromised such that Na_2_EDTA solution can penetrate to dissolve about 7.5% ^w^/_w_ of the iron in the core of the premix. The values were averages of 5 replicates ± standard deviation; the differences between means were considered significant at P < 0.05

**Fig. 3:**
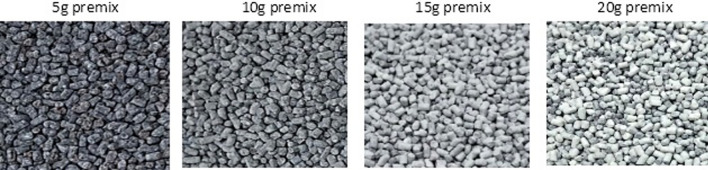
The SEM micrograph of premix samples coated with a suspension of TiO_2_ in soy stearin in a single pan-coating step. As the amount of TiO_2_ used for color masking increased, the compactness of TiO_2_ on the premix surface increased until 20% TiO_2_ was used. With 20% TiO_2_, 10% soy stearin's capacity to hold TiO_2_ in position on premix surface was compromised, such that TiO_2_ was falling off from the premix surface. The red arrow indicates particles of TiO_2_ that fell off from the surface of the premix

The micrograph of the premix coated with a suspension of TiO_2_ in soy stearin solution confirms some of the early results. The SEM images with X2700 magnification clearly showed a dispersed distribution of TiO_2_ in premixes coated with the suspension with lower amounts of TiO_2_ (5 and 10% ^w^/_w_, Fig. 3). The dispersed distribution is responsible for the lesser degree of whiteness observed. This distribution TiO_2_ became denser as the amount of TiO_2_ in the coating mixture increases. The SEM image at X35 magnification showed that TiO_2_ shed off from the premix coated with a suspension of 20% ^w^/_w_ TiO_2_ in 10% ^w^/_w_ soy stearin solution (indicated by red arrows in Fig. 3). This was not observed in the other three samples. Again, this suggests the suspension of 15% ^w^/_w_ TiO_2_ in 10% ^w^/_w_ soy stearin solution as the optimal coating formulation.

Although the premix was designed to disintegrate with most cooking methods, iron's bioaccessibility from the premix was evaluated for the very few, if any, cooking methods that may not disintegrate the premix before ingestion. While about 75% iron release was achieved with the premix that was color masked (15% ^w^/_w_ TiO_2_) and coated (10% ^w^/_w_ HPMC) after 30 min, this was not achieved in the premix coated with a suspension of TiO_2_ in soy stearin until after 90 min. Aside from this difference in the iron release profile, the two premix samples released about 80% ^w^/_w_ of their iron content after 2 h. This result suggests that even if the premix is not disintegrated during cooking, the iron in the premix should still be available for metabolic function once ingested.

### Stability of iodine in the salt formulated with the iron premix

Given the positive result obtained from the physical characteristics of the iron premix coated with a suspension of TiO_2_ (15% ^w^/_w_) in soy stearin (10% ^w^/_w_), the impact of this coat to prevent the adverse interaction between iron and iodine in the Double Fortified Salt was compared with the coat made with previous method (color masking first, then coated with soy stearin). Over 90% of the iodine added to the two salt samples was retained after six months of storage, even at 45 °C, 60–70% RH. Furthermore, there was no significant difference in iodine's stability in these salt samples, which showed that the 10% soy stearin coat with 15% TiO_2_ suspended in it prevented the moisture aided iodine degradation that may not be effectively prevented by an HPMC coat as suggested by Modupe (2020).

## Conclusions

The dark spot on the surface of iron premix caused by recycled and contaminated TiO_2_ was eliminated by coating with a suspension of 15% ^w^/_w_ TiO_2_ in soy stearin or HPMC (10% ^w^/_w_). Soy stearin outperformed HPMC in terms of color of the premix; being hydrophobic, it could better prevent the moisture aided iodine loss in the fortified salt. The premix made with this method has the same physical characteristics and ability to prevent adverse iron and iodine interaction in Double Fortified Salt as those made with the previous method. Over 90% of iodine added to the Double Fortified Salt was retained after 6 months, even at 45 °C, 60–70% RH. The new coating method ensures no need for double coating of the premix with HPMC and soy stearin. More so, this coating method simplifies the premix-making process by combining the previous method's color masking and coating step. The new coating formulation is achievable at the pilot plant as the drum coater's configuration can accommodate the new coating formulation.
